# Supplementary material for: Epigenetic loss of heterogeneity from low to high grade localized prostate tumours
Source: Nat Commun. 2021 Dec 15;12:7292. doi: 10.1038/s41467-021-27615-8 (PMC8674326; doi:10.1038/s41467-021-27615-8)
Supplement: Supplementary file 1 — Supplementary Information [file 41467_2021_27615_MOESM1_ESM.pdf]

A

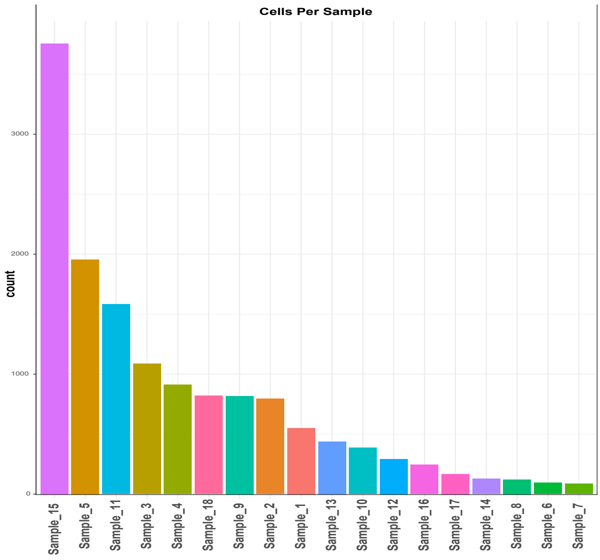

C

Descriptive statistics

Sample Name Paper	*Row*	*Total Number of Barcodes*	*Median Number of Sequencing Fragments*	*Median Number of Uniquely Mapped Fragments*	*Median Number of Mappability Ratio*	*Median Number of Properly Paired Ratio*	*Median Number of Duplicate Ratio*	*Median Number of ChrM Ratio*
**NA**	**AllSamples December.lda**	**14242**	**13436**	**1287.5**	**0.87**	**1**	**0.86**	**0.02**
Sample_1	G33_1	551	30893	1102	0.86	1	0.96	0.05
Sample_2	G33_2	796	10402.5	1998	0.89	1	0.78	0.03
Sample_3	G4_1	1087	70051	1803	0.91	1	0.97	0.02
Sample_4	G4_2	912	22114	3374	0.89	1	0.84	0.01
Sample_7	ID54773	86	3740.5	725	0.87	1	0.76	0.1
Sample_6	ID56893	95	8705	648	0.88	1	0.92	0.09
Sample_5	G33_3	1956	13263.5	3337	0.88	1	0.72	0.04
Sample_8	TB12146	122	34111.5	640.5	0.78	0.88	0.97	0.02
Sample_9	TB4732	819	7759	898	0.82	1	0.86	0.03
Sample_10	TB5020	387	9242	1209	0.87	1	0.86	0.02
Sample_11	TB6475	1582	7102.5	825	0.87	1	0.87	0.02
Sample_12	TB7521	293	5766	665	0.87	1	0.86	0.04
Sample_13	TB7677	439	18007	2229	0.83	1	0.87	0.1
Sample_14	TB7799	128	39672.5	714	0.8	0.87	0.98	0.02
Sample_15	TB9679	3757	10760	1358	0.88	1	0.87	0.01
Sample_16	WM306009	244	42762	673.5	0.77	0.88	0.98	0.05
Sample_17	WM306010	167	33514	644	0.79	0.89	0.97	0.03
Sample_18	WM306016	821	38961	704	0.81	0.89	0.97	0.04

B

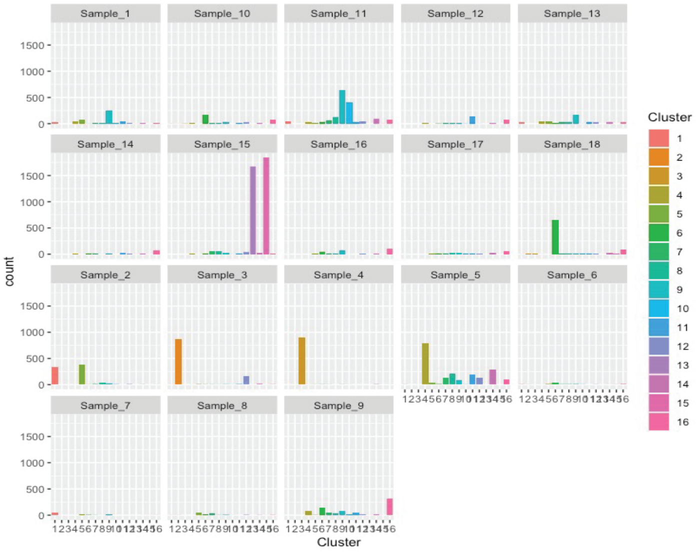

Supplementary Figure 1: 14,251 cells were sequenced from 18 prostate tumour samples. A. Number of cells sequenced per sample is shown. B. Distribution of cells from each tumour sample into different clusters. C. All DNA libraries were sequenced to near saturation as shown by median duplication rates. Quality measurements of the sci-ATAC-seq libraries produced in the study are shown, TotalNumberOfBarcodes: Total Number of Cells, MedianNumOfSequencingFragments: Median Number of Sequencing Fragments Per Cell, MedianNumberOfUniquelyMappedFragments: MedianNumberOfUniquelyMappedFragments, MedianNumberOfMappabilityRatio: Median Mapping Ratio Per Cell, MedianNumberOfProperlyPairedRatio: Median Ratio of Reads With A Paired Read Per Cell, MedianNumberOfDuplicateRatio: Median Ratio of Duplicates Per Cell, MedianNumberOfChrMRatio: Median Ratio of ChrM Mapped Reads Per Cell, Sample: Sample cistopic Name, SampleNamePaper: Sample Name Paper.

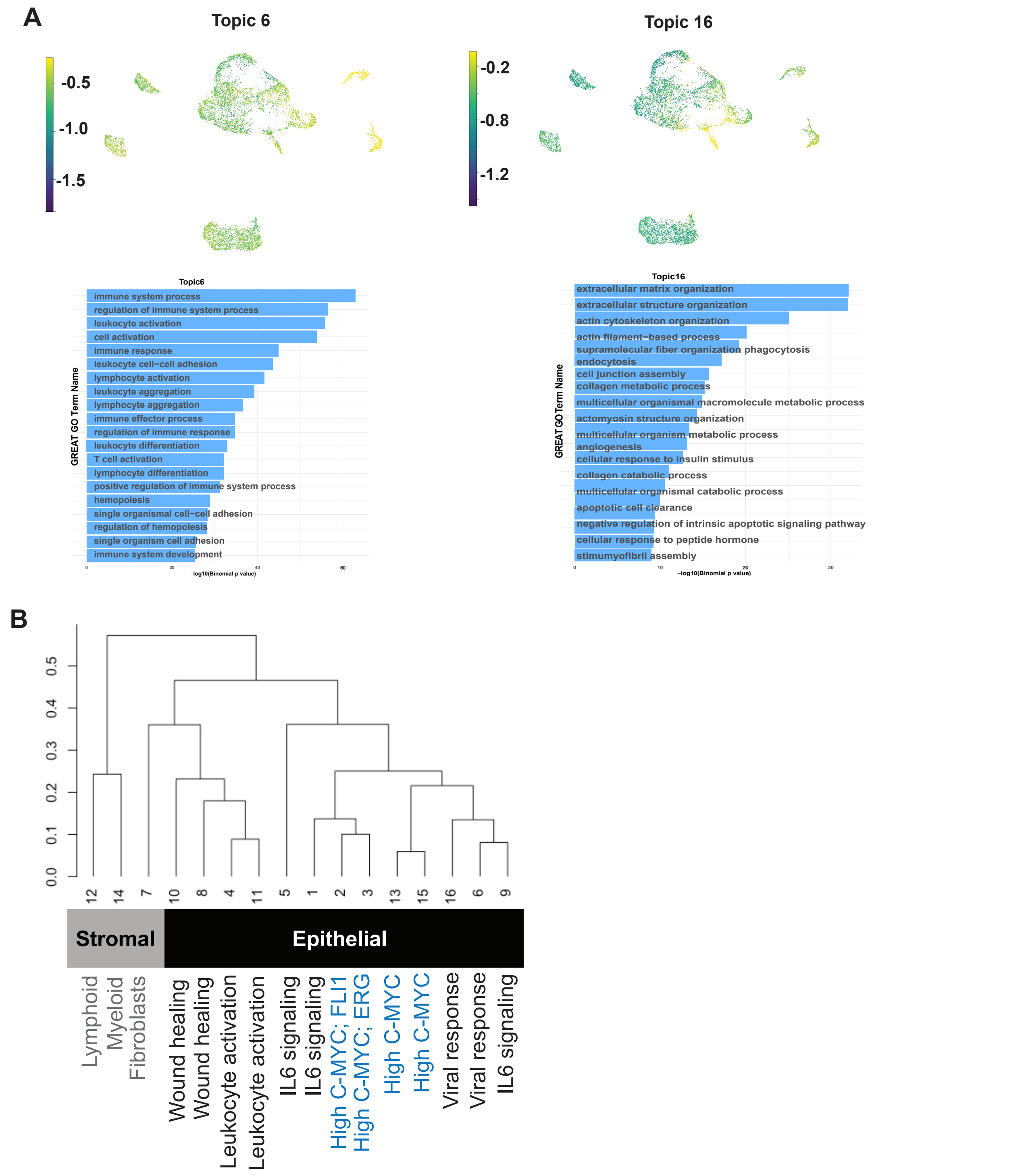

Supplementary Figure 2: Annotation of clusters using inferred gene expression. A. UMAP showing Topic 20 was highly enriched in cells within Cluster 14 (top), Topic 6 was highly enriched in cells within Cluster 12 (middle) and Topic 16 was highly enriched in cells within Cluster 7 (bottom). GREAT analysis of GO profiles (right) for each Topic identified Cluster 14 as myeloid immune, Cluster 12 as lymphoid immune and Cluster 7 as stromal cells. B. 16 Clusters that were identified around 30 Topics exhibit hierarchical clustering as shown by a cluster dendrogram. Clusters 12 and 14 consisted of immune cells, which formed a separate cluster in the dendrogram. Cluster 7 consisted of stromal cells associated with the prostate cancer epithelial cells. Clusters in the main island are enriched for GO terms associated an inflammatory response: wound healing (Clusters 8, 10), leukocyte activation (Cluster 11), viral response (Clusters 4, 6, 9, 16) and IL6 signalling (Clusters 1, 5). Outer clusters show high accessibility for C-MYC (Clusters 2, 3, 13, 15). Cluster 2 show accessibility for FLI1 and Cluster 3 for ERG.

A

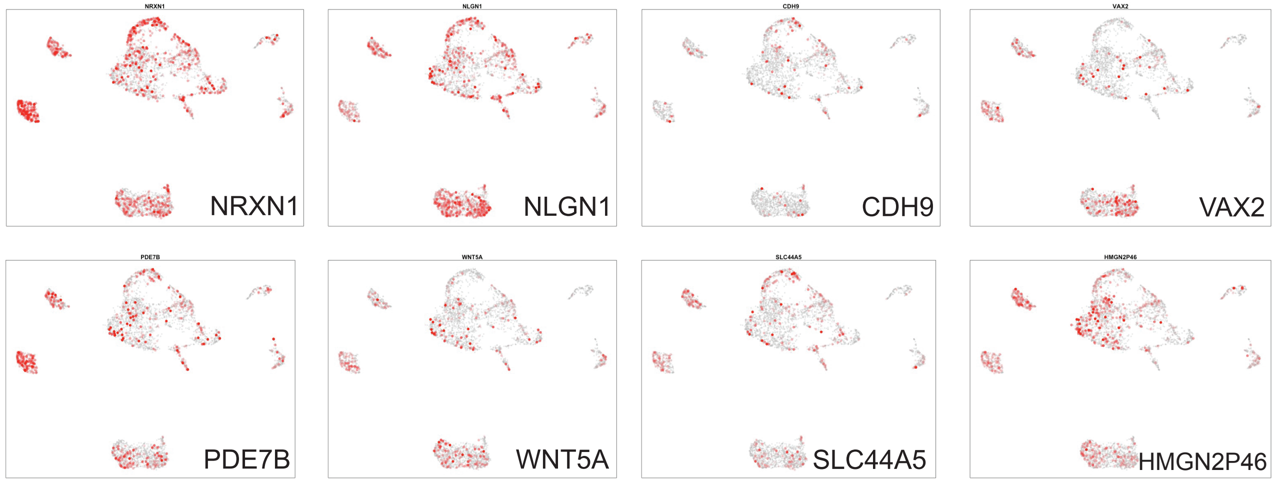

B

Gene	Region (distance to TSS)
CDH9	**+363,645**, +389,447, +460,265, +694,525, +695, 043, +791,034, +792,862, **+856,177**
NLGN1	**+793,177**
NRXN1	-732,656, -710,792, **-451,548**, **-405,520**, -255,085, **-227,262**, -47,803, **-32,947**, -4,865, **+87**, **+6,150**, **+68,490**, +321,293, +381,839, +426,956, +502,984, +695,379, +989,586

C

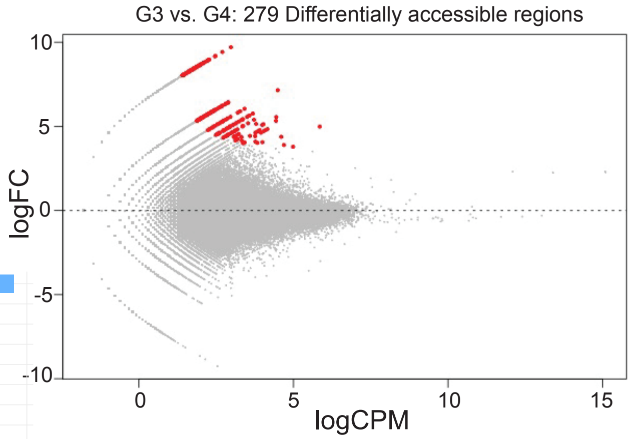

D

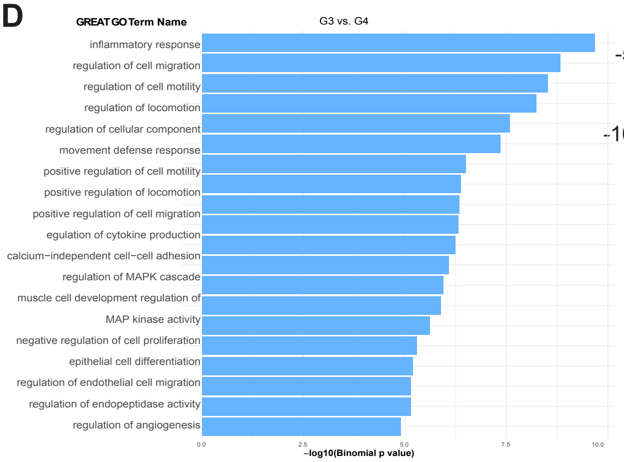

Supplementary Figure 3: The top differentially accessible chromatin regions identified in prostate tumours. A. Cells that have chromatin accessibility around the promoter of differentially accessible genes, NRXN1, NLGN1, CDH9, VAX2, PDE7B, WNT5A, SLC44A5 and HMGN2P46, selected from the top 23 regions are shown in red on the UMAP. B. Top peaks identified near neuronal adhesion molecules when Sample_15 is omitted from differential analysis. Peaks that are shared when Sample_15 is included in analysis are shown in bold. C. The top 279 differentially accessible chromatin regions are identified in prostate tumours with Gleason pattern 3 versus 4 (marked with red). D. GREAT GO results for accessible DNA elements in prostate tumours with Gleason pattern 3 are enriched for early inflammatory response.

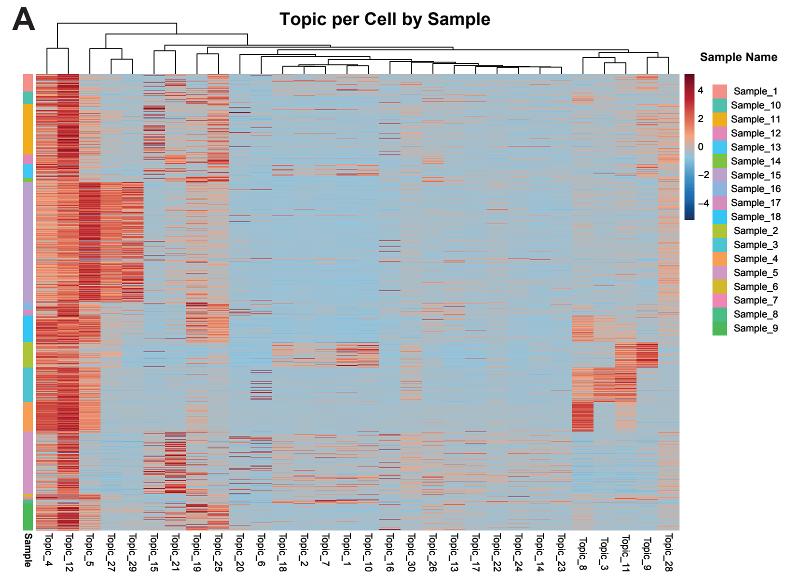

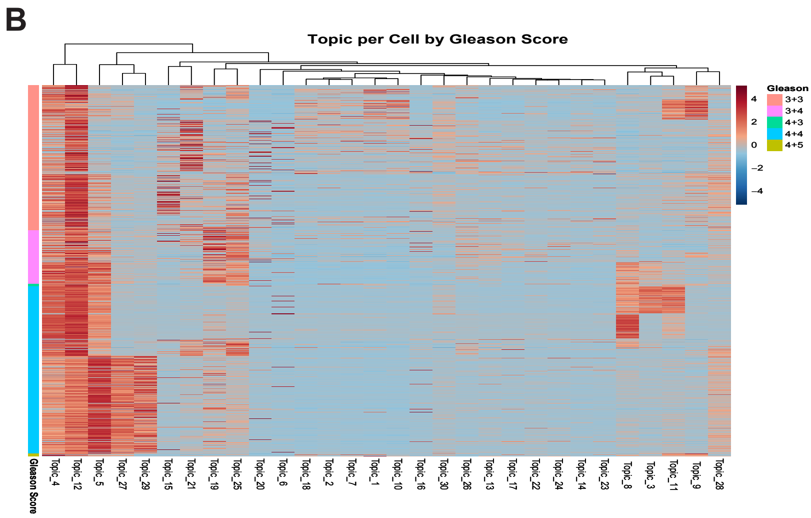

Supplementary Figure 4: Distinct Topics exist in Clusters that define tumours with Gleason pattern 3 and 4. A. Heatmap showing Topic enrichment in each sample. B. Heatmap showing the distribution of topics across different Gleason scores identified common and distinct pathways associated with Gleason pattern 3 and 4 tumours. C. GREAT GO results for Topics 3, 4, 16, 27 and 29 that are associated with Clusters defined in the study.

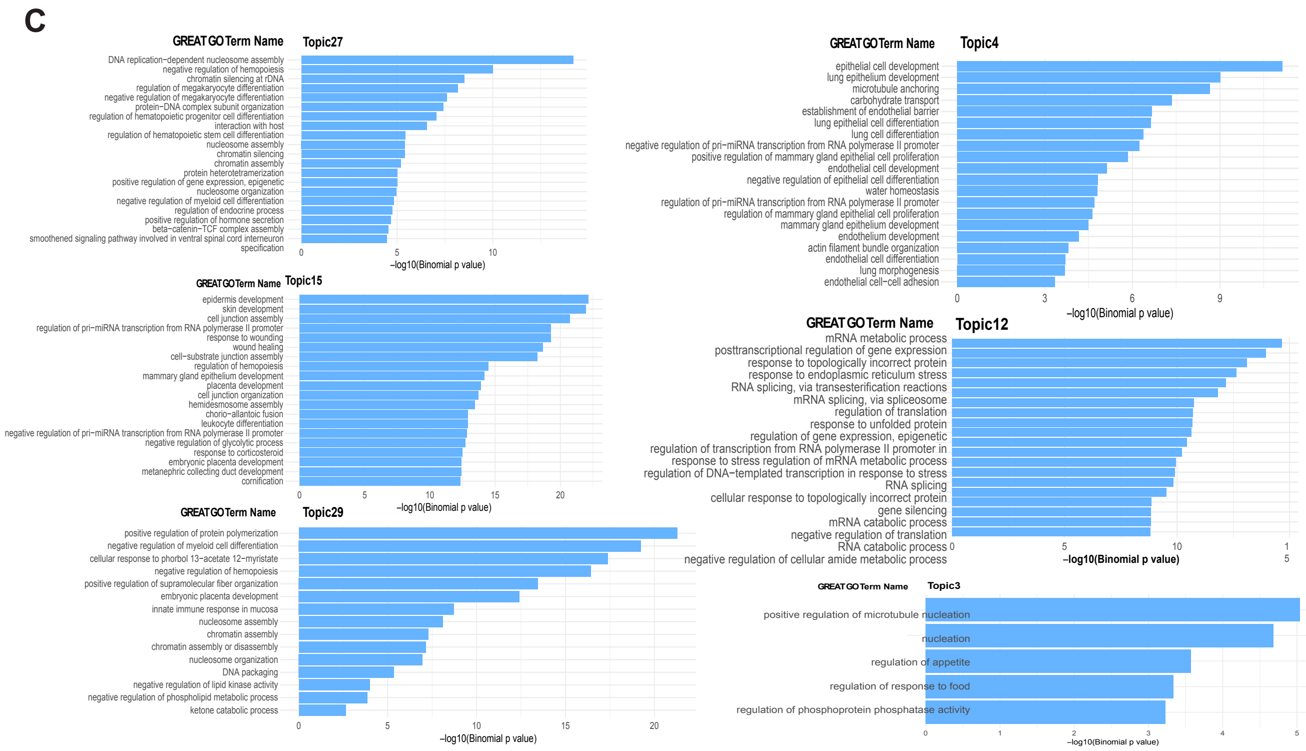

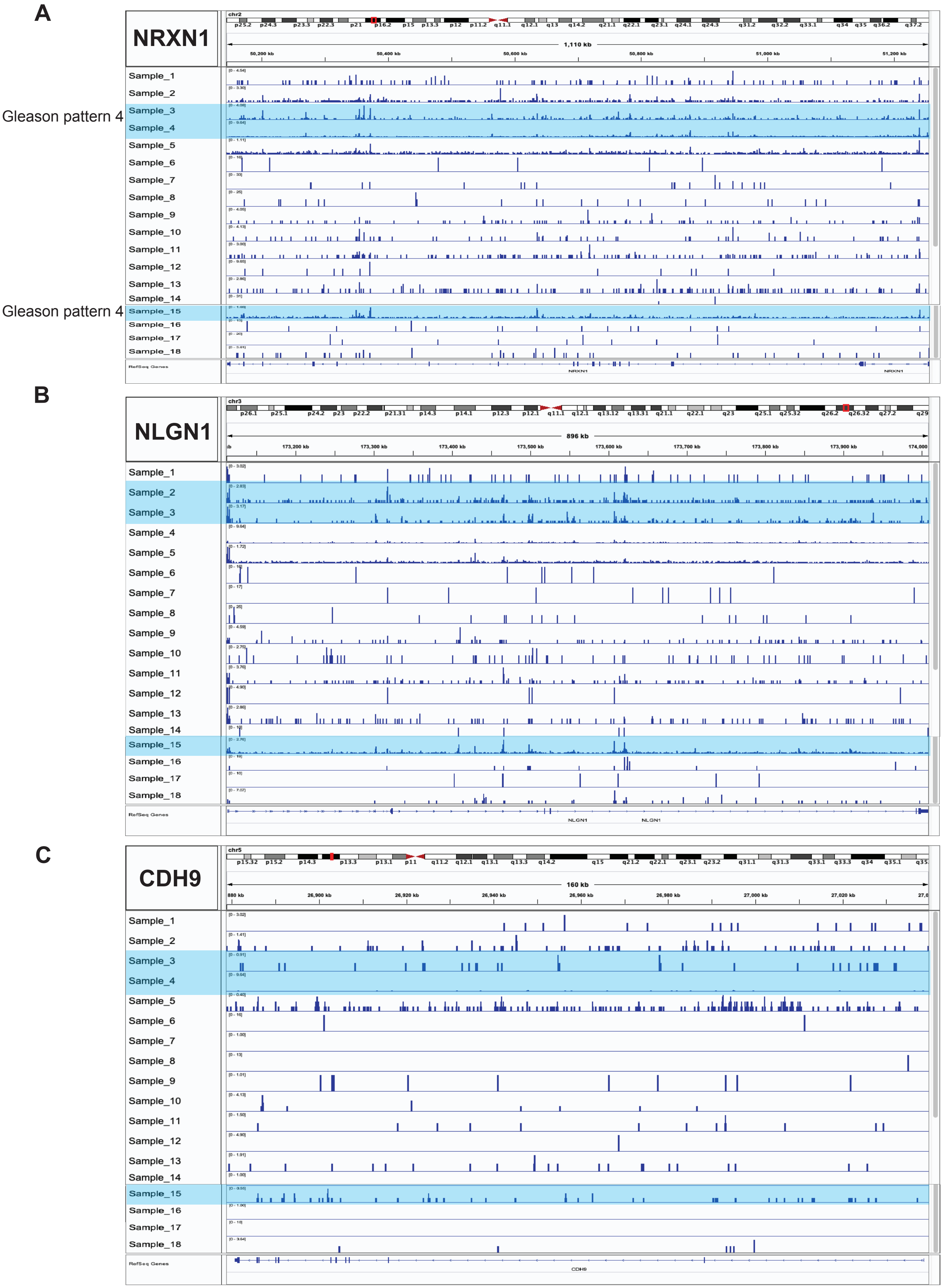

Supplementary Figure 5: Genome browser tracks showing peaks at the NRXN1 (A), NLGN1 (B) and CDH9 (C) loci. Samples with primary Gleason pattern 4 tumours are highlighted in blue.

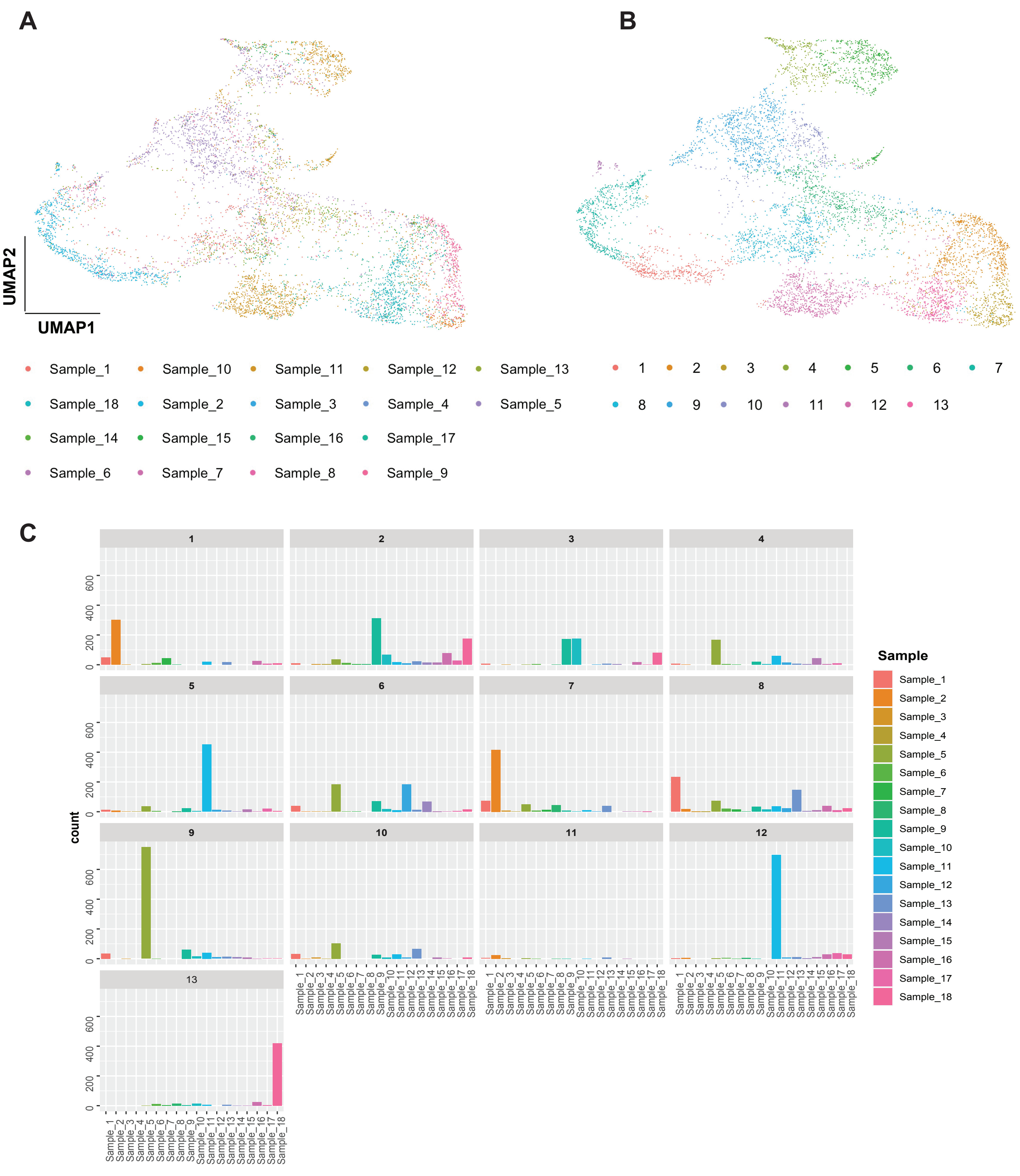

Supplementary Figure 6: Gleason pattern 3 cells do not form patient-specific clusters when analysed independently from Gleason pattern 4 cells. A. Single-cells from prostate samples were clustered using cisTOPIC based on their chromatin accessibility profiles. Cells are coloured according to their sample IDs on the UMAP. B. Single-cells from prostate samples were clustered using cisTOPIC based on their chromatin accessibility profiles. Cells are coloured according to their cluster IDs on the UMAP. C. Distribution of cells from each tumour sample into different clusters.

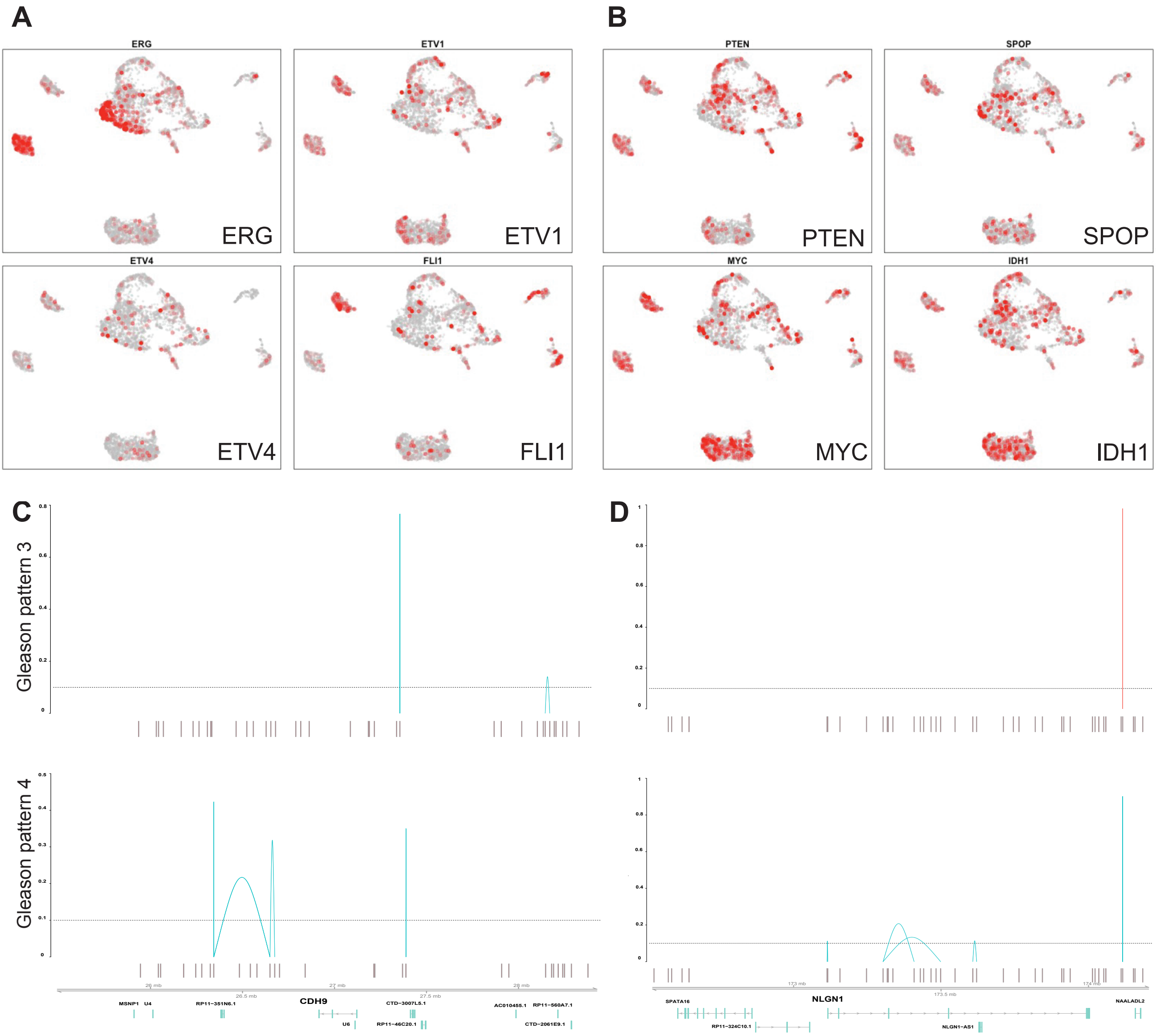

Supplementary Figure 7: Chromatin accessibility profiles of genes associated with known prostate cancer subtypes are enriched in specific clusters. A. ERG, ETV1, ETV4 and FLI1 accessibility are shown in red. Samples 4 and 18 show high accessibility for ERG whereas Sample 3 showed high accessibility to FLI1 and ETV1. B. PTEN, SPOP, MYC and IDH1 accessibility are shown in red. Higher accessibility for MYC is observed in tumours with Gleason pattern 4 (outer class). C-D. Cicero maps show higher number of putative regulatory interactions around the CDH9 (C) and NLGN1 (D) loci in Gleason pattern 3 (top) vs 4 (bottom) tumours.

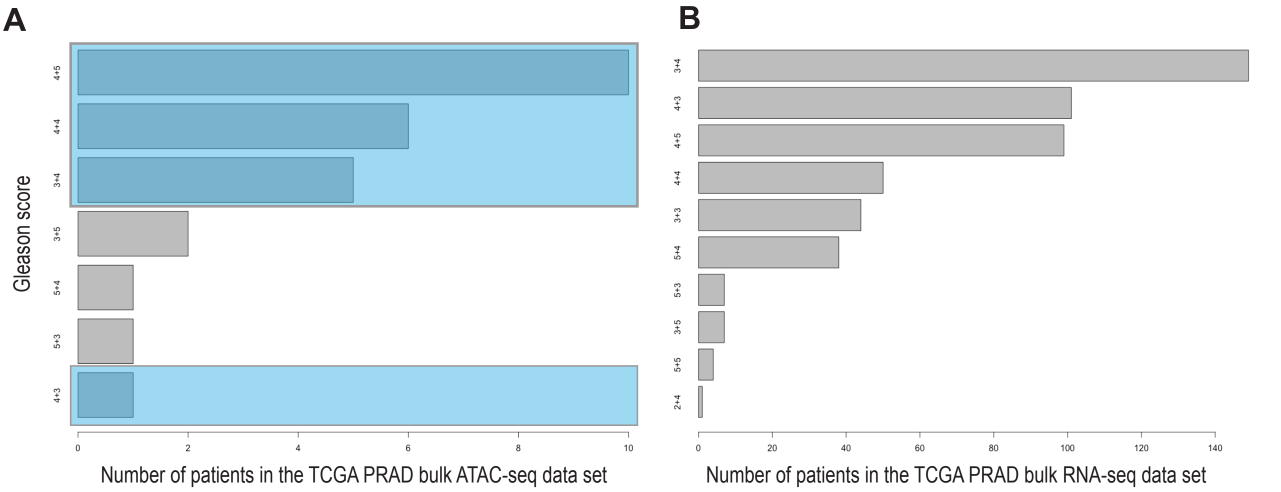

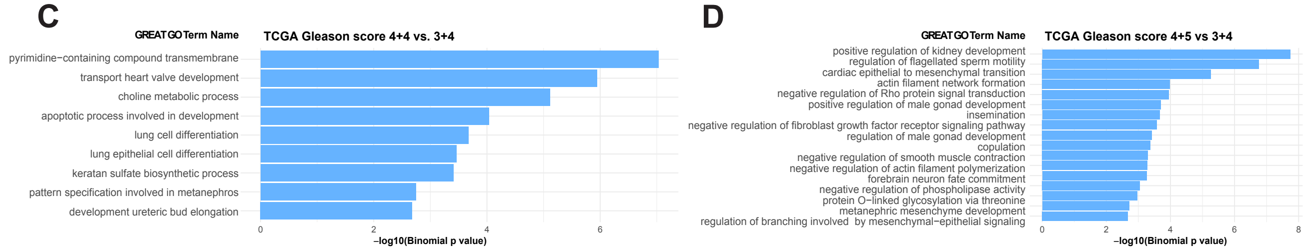

Gene	Region (distance to TSS)
CDH9	-349,114
NRXN1	-299,192, +635,988, +695,594, +989,463

Gene	Region (distance to TSS)
CDH9	-868,089, -641,977, -349,114
NRXN1	-732,627, -33,053, +695,594, +989,463

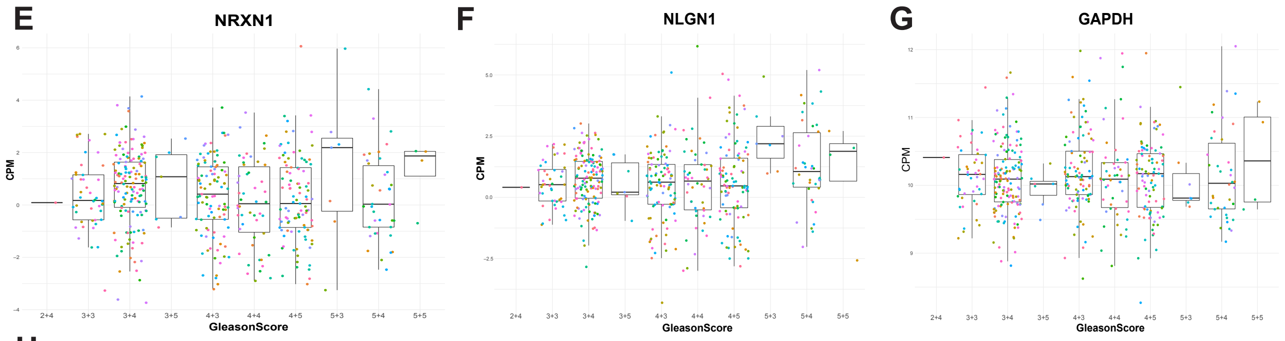

H

Gene	Sci-ATAC-seq	TCGA bulk ATAC-seq	Bp difference
NRXN1	Peak_59581 (-732656)	PRAD_13077 (-732627)	29
NRXN1	Peak_59566 (-126050)	PRAD_13075 (-126002)	48
NRXN1	Peak_59565 (-47803)	PRAD_13074 (-47727)	76
NRXN1	Peak_59553 (+313937)	PRAD_13071 (+314042)	105
NRXN1	Peak_59551 (+381839)	PRAD_13070 (+382023)	184
NRXN1	Peak_59550 (+388931)	PRAD_13069 (+389132)	201
NRXN1	Peak_59541 (+539682)	PRAD_13067 (+539723)	41
NRXN1	Peak_59540 (+553909)	PRAD_13066 (+554264)	355
NRXN1	Peak_59539 (+615053)	PRAD_13064 (+615206)	153
NRXN1	Peak_59538 (+624088)	PRAD_13063 (+623999)	89

Gene	Sci-ATAC-seq	TCGA bulk ATAC-seq	Bp difference
NRXN1	Peak_59536 (+681698)	PRAD_13062 (+681930)	232
NRXN1	Peak_59535 (+695379)	PRAD_13061 (+695594)	215
NRXN1	Peak_59531 (+888100)	PRAD_13060 (+887952)	148
NRXN1	Peak_59530 (+898213)	PRAD_13059 (+898451)	238
NRXN1	Peak_59529 (+905336)	PRAD_13058 (+905423)	87
NRXN1	Peak_59528 (+910520)	PRAD_13057 (+910520)	0
NRXN1	Peak_59526 (+960054)	PRAD_13056 (+960137)	83
NRXN1	Peak_59524 (+989586)	PRAD_13055 (+989463)	123
CDH9	Peak_90760 (-349238)	PRAD_31624 (-349114)	124
CDH9	Peak_90749 (+389446)	PRAD_31621 (+389332)	114
CDH9	Peak_90739 (+792862)	PRAD_31619 (+793047)	185

Supplementary Figure 8: NRXN1 and CDH9 are significantly accessible in tumours with Gleason score 4+4 and 4+5 as compared to Gleason score 3+4 in the TCGA bulk ATAC-seq data set. A. Distribution of Gleason scores across patients are shown in the TCGA bulk ATAC-seq data set. x-axis shows the number of patients in the cohort, y-axis indicates the Gleason score. Patient samples with primary Gleason grades 3 and 4 used in differential analysis are highlighted in blue. B. Distribution of Gleason scores across patients are shown in the TCGA RNA-seq data set. C. GREAT GO results (top) for TCGA bulk ATAC-seq data for prostate tumours with Gleason pattern 4 (Gleason score 4+4) as compared to tumours predominantly Gleason pattern 3 (Gleason score 3+4). Identified peaks that are linked to CDH9 and NRXN1 are shown (bottom). D. GREAT GO results (top) for TCGA bulk ATAC-seq data for prostate tumours with predominantly Gleason pattern 4 (Gleason score 4+5) as compared to tumours predominantly Gleason pattern 3 (Gleason score 3+4). Identified peaks that are linked to CDH9 and NRXN1 are shown (bottom). E-G. Bulk RNA-seq results from 497 patients are shown (TCGA's PRAD data set), each dot representing a patient. Patients are categorized into columns based on their Gleason scores as indicated on the x-axis. Gleason score 2 +4=1 patient; Gleason score 5+5=4 patients; Gleason score 3+5=7 patients; Gleason score 5+3=7 patients; Gleason score 5+4=38 patients; Gleason score 3+3=44 patients; Gleason score 4+4=50 patients; Gleason score 4+5=99 patients; Gleason score 4+3=101 patients; Gleason score 3+4=146 patients. Quartiles are 25%, 50% and 75% and 50% shows the median. Interquartile range is the difference between the 75th and 25th percentile. The upper whisker is the maximum value of the data that is within 1.5 times the interquartile range over the 75th percentile. The lower whisker is the minimum value of the data that is within 1.5 times the interquartile range over the 25th percentile. Outlier values are considered any values over 1.5 times the interquartile range over the 75th percentile or under the 25th percentile. Fragments Per Kilobase of transcript per Million mapped reads (FPKM) are shown on the y-axis. NRXN1 expression is shown across patients with different Gleason scores (E). NLGN1 expression is shown across patients with different Gleason scores (F). GAPDH (control) expression is shown across patients with different Gleason scores (G). H. The list of peaks at NRXN1 and CDH9 loci from the TCGA bulk ATAC-seq data set that match with the peaks in our data set (sci-ATAC-seq). The third column shows the base pair difference between each peak.

A

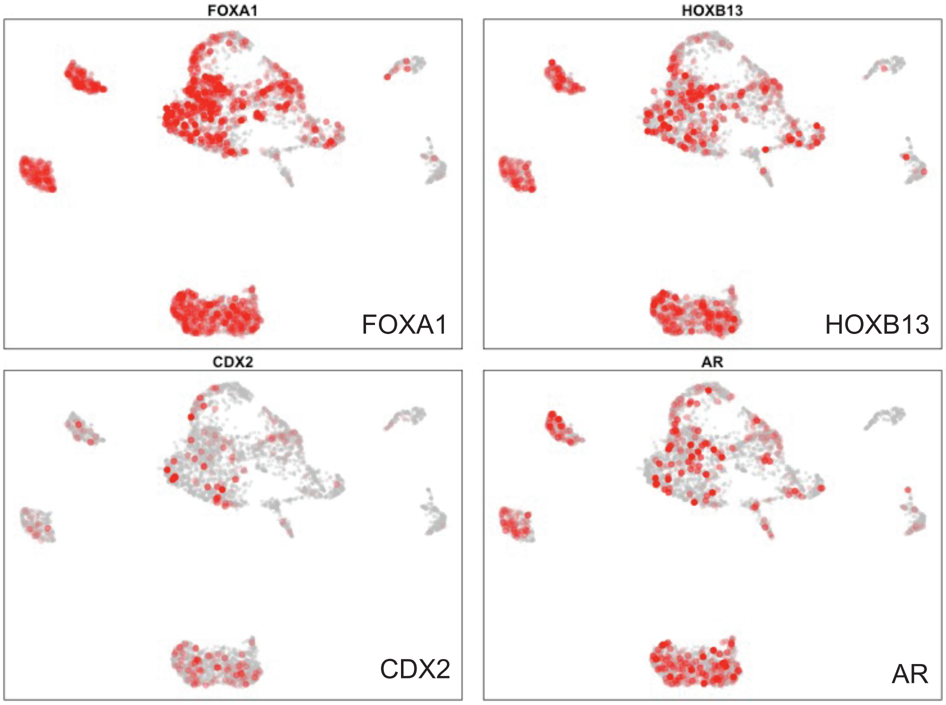

B

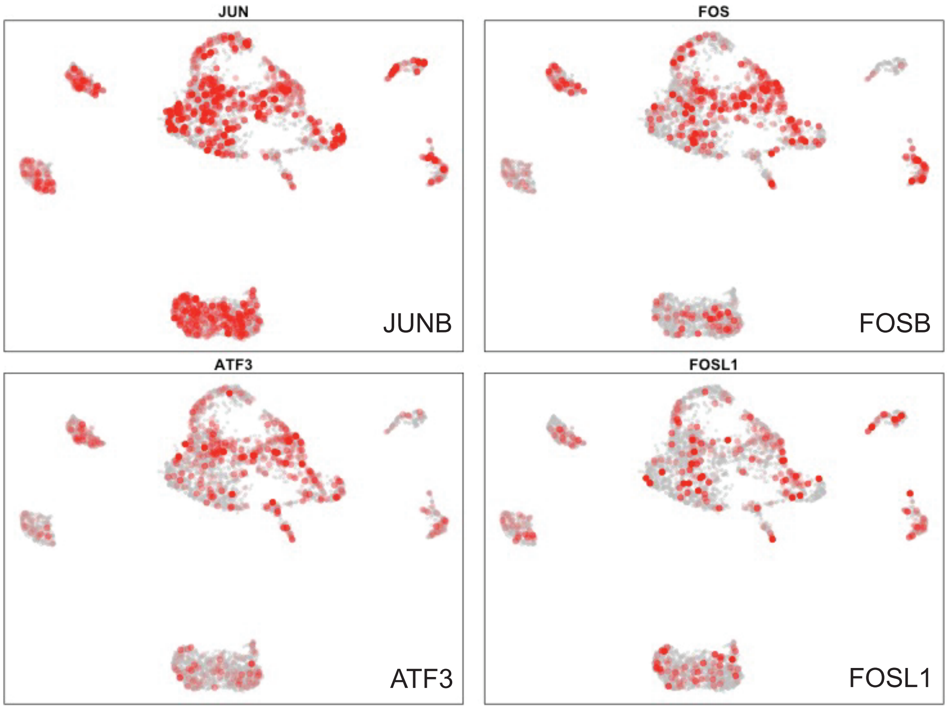

Supplementary Figure 9: Chromatin accessibility profiles of specific transcription factors are enriched in prostate tumours with Gleason pattern 3 and 4. Cells that have accessibility for the promoter region of the gene of interest are shown in red. A. Accessibility to the promoter region of TFs FOXA1, HOXB13, CDX2 and AR are shown. B. Accessibility to the promoter region of JUN, FOS, ATF3 and FOSL1 are shown.

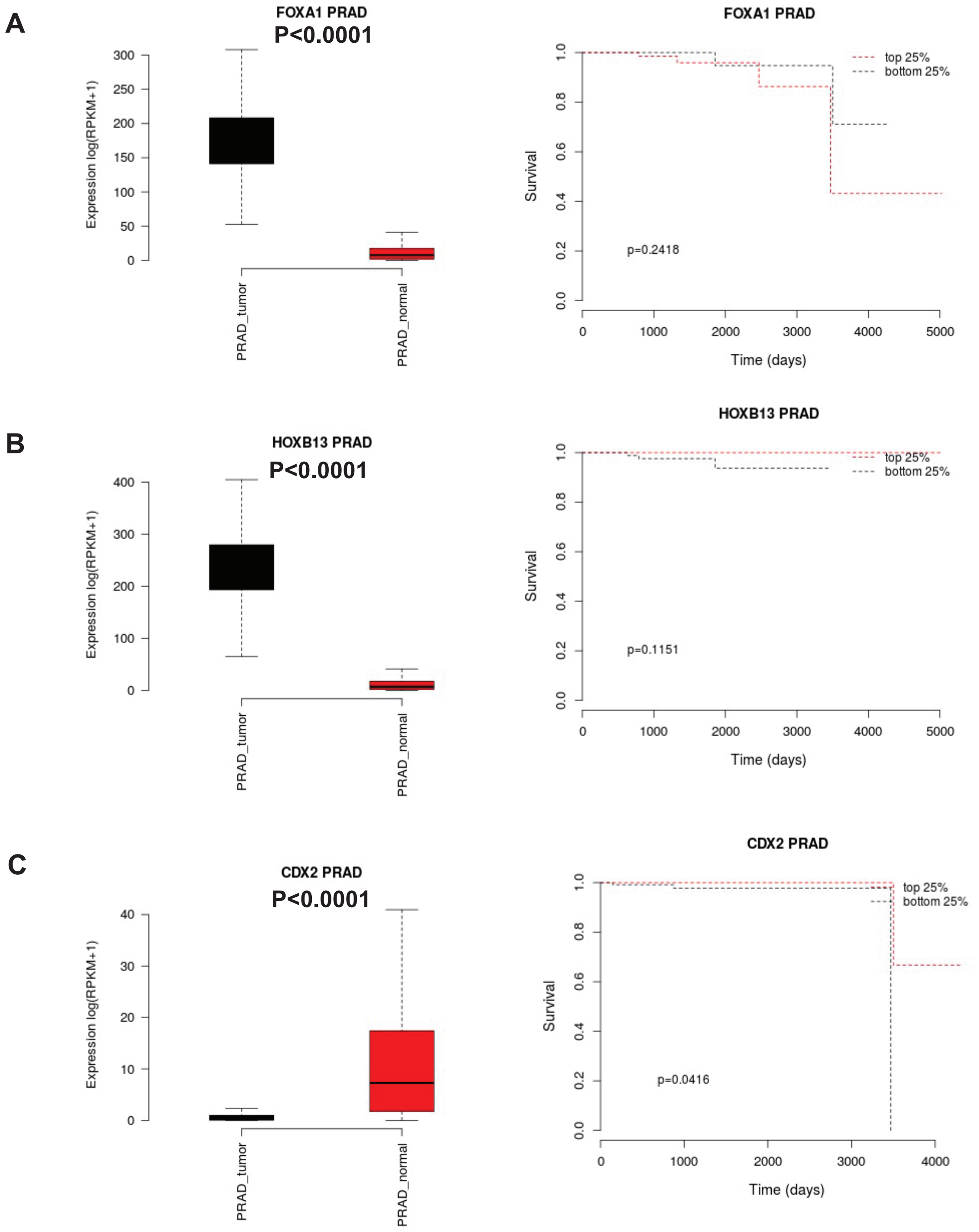

Supplementary Figure 10: TCGA data from 333 primary prostate tumors and 19 tumor adjacent normal samples show the link between the expression levels of TFs FOXA1, HOXB13 and CDX2 and patient survival. A. FOXA1 expression in prostate adenocarcinoma (PRAD) tumours (black) and normal (red) tissue (P<000.1). Patient survival (right) data is shown for the top 25th percentile of patients (red line) and the bottom 25th percentile (black line). B. HOXB13 expression in PRAD tumours (black) and normal (red) tissue (P<000.1). Patient survival (right) data is shown for the top 25th percentile of patients (red line) and the bottom 25th percentile (black line). C. CDX2 expression in PRAD tumours (black) and normal (red) tissue (P<000.1). Patient survival (right) data is shown for the top 25th percentile of patients (red line) and the bottom 25th percentile (black line). Error bars show SD values for box plots. P-values are calculated using Wilcox rank sum test for bar graphs. P-values are calculated using Kaplain-Meier log-rank test for survival plots.

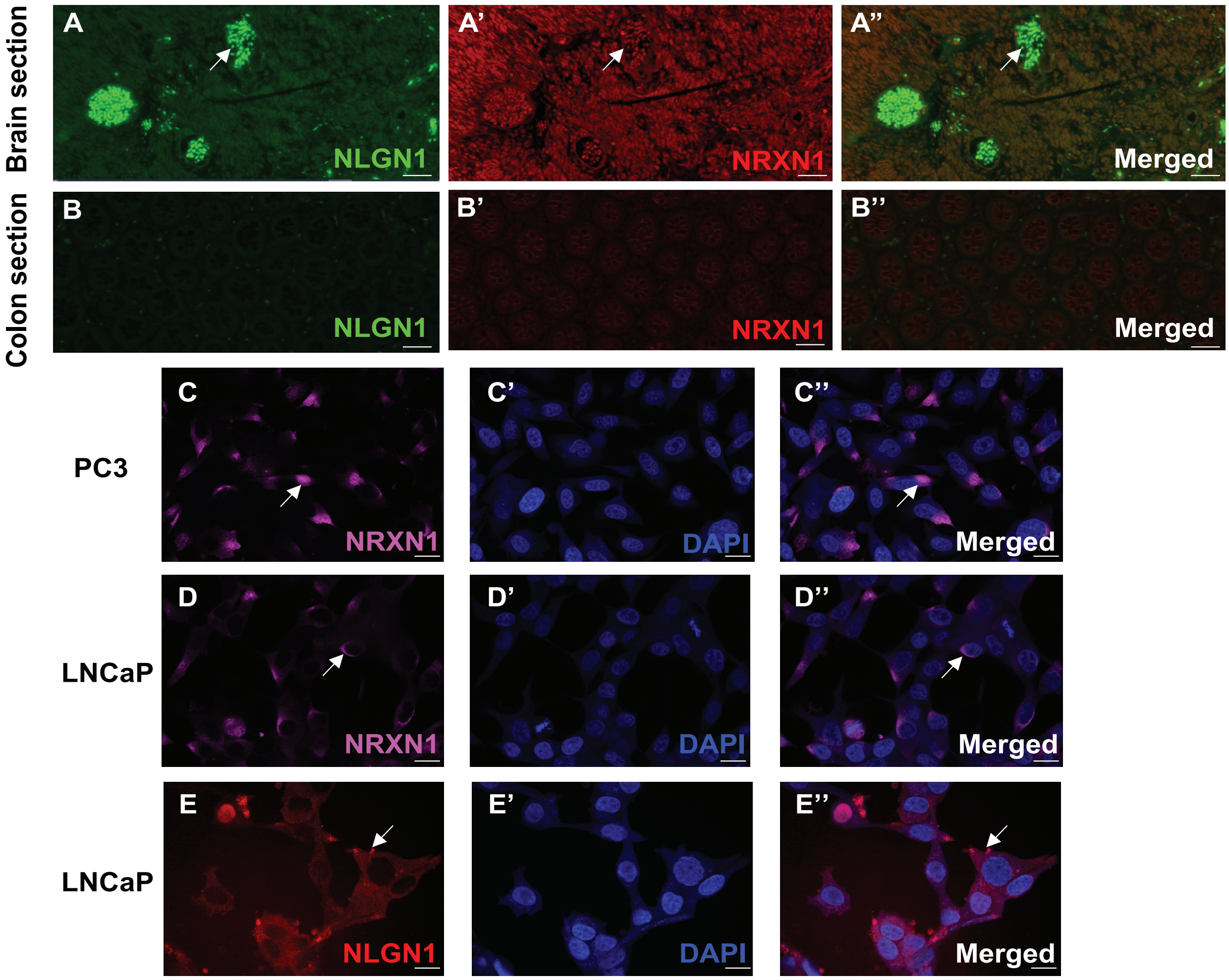

F

	AF488	AF555	AF647	AF750
1		NLGN1		NRXN1
2			NCAM	CK5
3	AF			
4		AR	CK8	ChrA
5		ECAD	CD31	CD3
6	AF			
7		ERG		CK14

Supplementary Figure 11: Antibodies against NRXN1 and NLGN1 were tested and validated using different tissue sections and cell lines for cyclic IF. A. Brain tissue section stained with the NLGN1 (green) and NRXN1 (red) as a positive control for the antibodies. White arrows show an example region with positive NLGN1 and NRXN1 expression. Scale bar (white line) shows 200 pixels (n=1) B. Colon tissue section stained with the NLGN1 (green) and NRXN1 (red) as a negative control for the antibodies. Scale bar (white line) shows 200 pixels (n=1). C. NRXN1 antibody tested on fixed PC3 prostate cancer cells (C, magenta) shown in contrast to DAPI (C', blue). C'' shows the two channels merged. White arrow shows NRXN1 expression. Scale bar (white line) shows 20 µm (n=3). D. NRXN1 antibody tested on fixed LNCaP prostate cancer cells (D, magenta) shown in contrast to DAPI (D', blue). D'' shows the two channels merged. White arrow shows NRXN1 expression. Scale bar (white line) shows 20 µm (n=3). E. NLGN1 antibody tested on fixed LNCaP prostate cancer cells (E, magenta) shown in contrast to DAPI (E', blue). E'' shows the two channels merged. White arrow shows NLGN1 expression. Scale bar (white line) shows 20 µm (n=3). F. Rounds table for cyclic IF experiments show cell-type specific markers. Each column is marked by the Alexa Fluor (AF) dye that are attached to the antibodies within that column: AF488, AF555, AF647 and AF750. Each row shows the antibodies that are used in that round of antibody staining. Autofluorescence (AF) levels are measured at the end of round 2 and round 5. The cyclic IF experiment has been performed once with the list of markers indicated in the table for 8 tissue-sections.

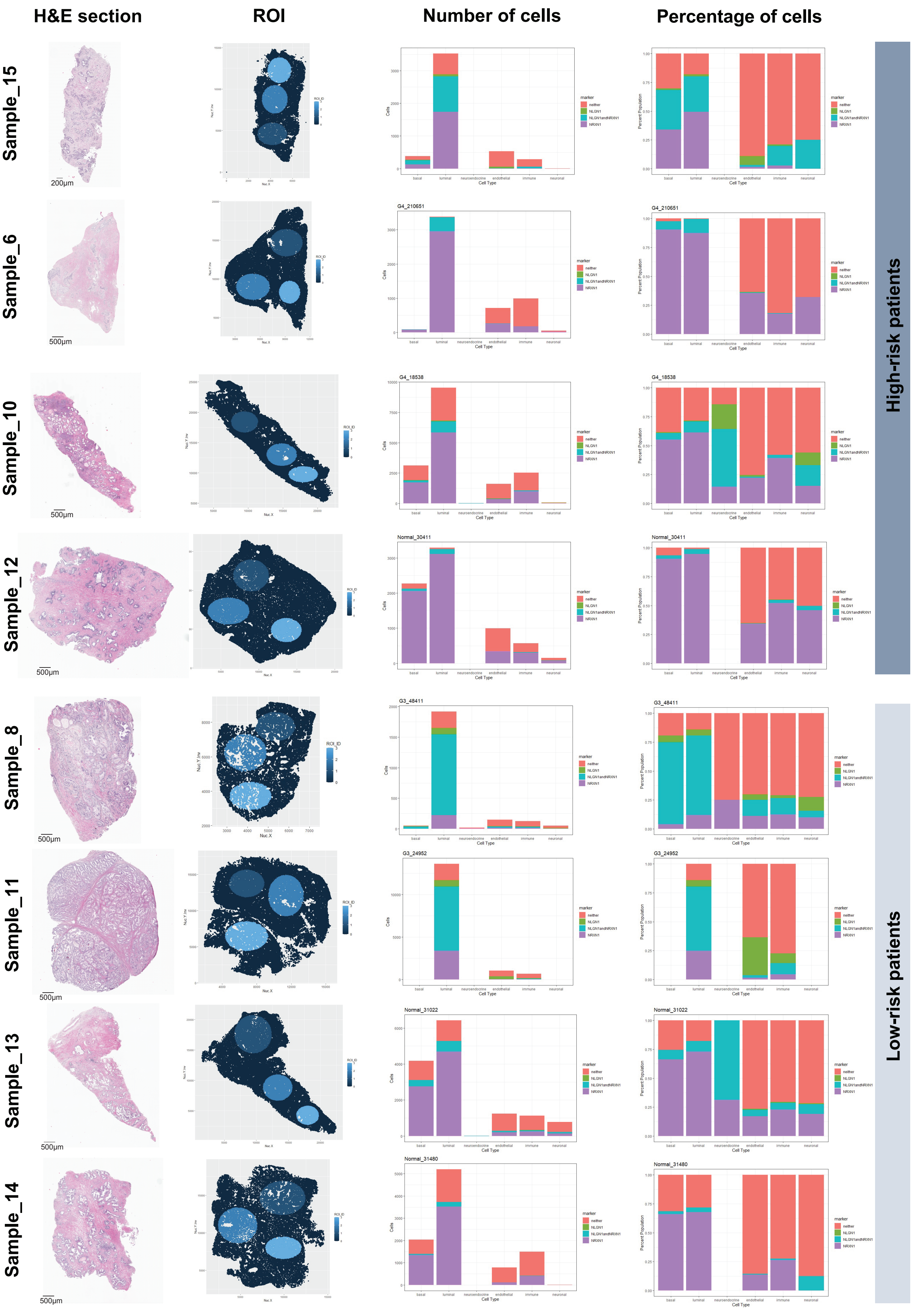
H&E section
ROI
Number of cells
Percentage of cells
Sample_15
Sample_6
Sample_10
Sample_12
Sample_8
Sample_11
Sample_13
Sample_14
High-risk patients
Low-risk patients
200µm
500µm
G4_210651
G4_18538
Normal_30411
G3_48411
G3_24952
Normal_31022
Normal_31480
Cells
Percent Population
Cell Type
basal
luminal
neuroendocrine
endothelial
immune
neuronal
marker
neither
NLGN1
NLGN1andNRXN1
NRXN1
ROI_ID
Nuc Y Inv
Nuc X

Supplementary Figure 12: NRXN1 and NLGN1 are expressed in tumours with Gleason pattern 3 and 4. H&E tissue sections from all patients are shown (left). Scale bars show 200μm (Sample_15) and 500μm. Three regions of interest (ROIs) were drawn in each whole-mount tissue section based on their H&E staining (blue diagrams). The expression of NRXN1 and NLGN1 are measured across different cell types within each ROI (bar graphs). x-axis shows cell types and y-axis shows the total number of segmented cells or percentage population of cells positive for that marker. NRXN1 expressing cells are shown in purple and NLGN1 in green. Cells that express both proteins are shown in blue and cells that do not express either are marked with pink.
