# Supplementary material for: Epigenetic loss of heterogeneity from low to high grade localized prostate tumours
Source: Nat Commun. 2021 Dec 15;12:7292. doi: 10.1038/s41467-021-27615-8 (PMC8674326; doi:10.1038/s41467-021-27615-8)
Supplement: Supplementary file 3 — Description of Additional Supplementary Files [file 41467_2021_27615_MOESM3_ESM.pdf]

Description of Additional Supplementary Files

File Name: Supplementary Data 1
Description: CAPRA-S and MSKCC nomogram calculations of patients in our cohort.

File Name: Supplementary Data 2
Description: CAPRA calculations of patients in the TCGA PRAD cohort.

File Name: Supplementary Data 3
Description: Tn5 and PCR oligo sequences used in the study.
