# Supplementary material for: Epigenetic loss of heterogeneity from low to high grade localized prostate tumours
Source: Nat Commun. 2021 Dec 15;12:7292. doi: 10.1038/s41467-021-27615-8 (PMC8674326; doi:10.1038/s41467-021-27615-8)
Supplement: Supplementary file 7 — Reporting Summary [file 41467_2021_27615_MOESM7_ESM.pdf]

nature research

Corresponding author(s): Sebnem Ece Eksi, Andrew C. Adey

Last updated by author(s): November 18, 2021

Reporting Summary

Nature Research wishes to improve the reproducibility of the work that we publish. This form provides structure for consistency and transparency in reporting. For further information on Nature Research policies, see our Editorial Policies and the Editorial Policy Checklist.

Statistics

For all statistical analyses, confirm that the following items are present in the figure legend, table legend, main text, or Methods section.

n/a	Confirmed	
☐	☒	The exact sample size (*n*) for each experimental group/condition, given as a discrete number and unit of measurement
☒	☐	A statement on whether measurements were taken from distinct samples or whether the same sample was measured repeatedly
☐	☒	The statistical test(s) used AND whether they are one- or two-sided<br>*Only common tests should be described solely by name; describe more complex techniques in the Methods section.*
☒	☐	A description of all covariates tested
☐	☒	A description of any assumptions or corrections, such as tests of normality and adjustment for multiple comparisons
☒	☐	A full description of the statistical parameters including central tendency (e.g. means) or other basic estimates (e.g. regression coefficient) AND variation (e.g. standard deviation) or associated estimates of uncertainty (e.g. confidence intervals)
☐	☒	For null hypothesis testing, the test statistic (e.g. *F*, *t*, *r*) with confidence intervals, effect sizes, degrees of freedom and *P* value noted<br>*Give P values as exact values whenever suitable.*
☒	☐	For Bayesian analysis, information on the choice of priors and Markov chain Monte Carlo settings
☒	☐	For hierarchical and complex designs, identification of the appropriate level for tests and full reporting of outcomes
☒	☐	Estimates of effect sizes (e.g. Cohen's *d*, Pearson's *r*), indicating how they were calculated

Our web collection on statistics for biologists contains articles on many of the points above.

Software and code

Policy information about availability of computer code

Data collection	FACSDiva v8.0.3 for BD FACSAria Fusion Cell Sorter, QuantStudio v1.7.1 for real-time PCR, NextSeq500 NCS v4.0 for sequencing, Zen 2 for imaging with Zeiss AxioScan.
Data analysis	FastQ files were generated from BCL files using bcl2fastq (Illumina Inc., v2.19.0). Fastq files were aligned, filtered, and analyzed using Snaptools v1.1 (https://github.com/r3fang/SnapTools) and scitools v2.0.2 (github.com/adeylab/scitools). Code used for the analysis of sci-ATAC-seq data in this study is available on Github (https://github.com/AlexChitsazan/ProstateTumorATACCode). GREAT (v4.0.4) was used for functional genomic region analysis. HOMER (v4.11) was used for transcription factor binding motif analysis. Cicero (v1.0.0) was used to predict cis-regulatory interactions. All czi files were converted into TIFF using the Zen 2 software. A custom software was used to register the nuclear DAPI images (Chang, 2017). Nuclei and cytoplasm segmentation were done using the QiTissue software (http://www.qi-tissue.com/). Code used for cyclic IF analysis in this study is available on GitHub (https://github.com/zeynepsayar/Neuronal_cyclic_R). Custom codes are deposited to Zenodo (https://zenodo.org/record/5644071#.YYLw7NbMI8M and doi:10.5281/zenodo.5635457).

For manuscripts utilizing custom algorithms or software that are central to the research but not yet described in published literature, software must be made available to editors and reviewers. We strongly encourage code deposition in a community repository (e.g. GitHub). See the Nature Research guidelines for submitting code & software for further information.

Data

Policy information about availability of data

All manuscripts must include a data availability statement. This statement should provide the following information, where applicable:

- Accession codes, unique identifiers, or web links for publicly available datasets
- A list of figures that have associated raw data
- A description of any restrictions on data availability

The raw and processed single-cell ATAC-sequencing files and processed data files are available in GEO under the super series GSE: GSE171559: https://www.ncbi.nlm.nih.gov/geo/query/acc.cgi?acc=GSE171559. TCGA bulk ATAC-seq data is publicly available here: https://gdc.cancer.gov/about-data/publications/ATACseq-AWG. TCGA bulk RNA-seq data is publicly available from gdc.cancer.gov under the accession code prad-2015 [https://gdc.cancer.gov/about-data/publications/prad_2015]. hg19 reference genome is available here: https://www.ncbi.nlm.nih.gov/assembly/GCF_000001405.39. We used the Human Protein Atlas as a reference for the expression pattern obtained from each antibody (https://www.proteinatlas.org/).

Field-specific reporting

Please select the one below that is the best fit for your research. If you are not sure, read the appropriate sections before making your selection.

- [x] Life sciences
- [ ] Behavioural & social sciences
- [ ] Ecological, evolutionary & environmental sciences

For a reference copy of the document with all sections, see nature.com/documents/nr-reporting-summary-flat.pdf

Life sciences study design

All studies must disclose on these points even when the disclosure is negative.

Sample size	For single-cell analysis, hundreds of individual profiles were obtained from similar size tumors. All patients meeting the clinical criteria with proper consent were included. We included at least seven patients of each clinical grade: primary Gleason pattern 3 (8 patient samples) and primary Gleason pattern 4 (7 patient samples). Same sizes were selected to be equal across clinical grades to enable proper representation. The number of single-cell libraries to be generated was selected based on required sequencing depths from previous publications on single-cell chromatin accessibility, as well as the analyses to be performed (https://doi.org/10.1038/ncomms15081; DOI: 10.7554/eLife.64090; https://doi.org/10.1093/hmg/ddab006).
Data exclusions	Reads were excluded if the indexes did not match a predetermined barcode sequence. Barcodes were filtered based on >=500 uniquely aligned reads and Q-score >= 10. Samples were also excluded if the single cell library resulted in less than 75 cells after previously stated filtering criteria.
Replication	The sci-ATAC protocol was used to generate a total of 7 library pools across 7 separate experiments. This demonstrates the reproducibility of the technique. The ability to reproduce single-cell chromatin accessibility profiles was demonstrated by co-clustering of single cells independent of library origin and the clustering analysis in which thousands of single cells showed strong cell type specific signal. For cyclic immunofluorescence experiments, 8 tissue-sections were stained with the same set of antibodies. Expression pattern of each antibody is consistent across all tissues, including positive and negative control tissue-sections, and our results recapitulate the known expression patterns as published by the Human Protein Atlas (https://www.proteinatlas.org/). As such, all attempts on replication for the immunofluorescence staining was successful.
Randomization	Randomization of conditions was performed for all experiments. For the first round of indexing, all conditions were tiled by column across each 96-well tagmentation plate. For the second round of indexing, all conditions were pooled and sorted into PCR plates, therefore, randomizing nuclei identity.
Blinding	As stated in the Randomization response above, nuclei are randomly sampled during the second indexing step in sci-ATAC. The nuclei identities within each reaction is unknown to the experimenter throughout library construction and sequencing. Reads were processed using the same pipeline without barcodes assigned to a specific cell line until final analysis. Patient samples that have different primary Gleason patterns were blindly mixed and tagged during combinatorial indexing. Non-hierarchical clustering was used to cluster all single-cells without taking into account their disease grades. Blinding was not relevant in the cyclic immunofluorescence experiment. Patient samples were grouped according to their disease grade.

Reporting for specific materials, systems and methods

We require information from authors about some types of materials, experimental systems and methods used in many studies. Here, indicate whether each material, system or method listed is relevant to your study. If you are not sure if a list item applies to your research, read the appropriate section before selecting a response.

Materials & experimental systems

n/a	Involved in the study	
☐	☒	Antibodies
☐	☒	Eukaryotic cell lines
☒	☐	Palaeontology and archaeology
☒	☐	Animals and other organisms
☐	☒	Human research participants
☒	☐	Clinical data
☒	☐	Dual use research of concern

Methods

n/a	Involved in the study	
☒	☐	ChIP-seq
☒	☐	Flow cytometry
☒	☐	MRI-based neuroimaging

Antibodies

Antibodies used	NRXN1, Millipore Sigma, Concentration (1:100), Cat# ABN161-I; RRID:AB_11211973; NLGN1, Millipore Sigma, Concentration (1:100), Cat# MABN742; CK5, Biolegend, Concentration (1:200), Cat# 905501; RRID:AB_2565050; CK8, Concentration (1:100), Abcam, Cat# ab192468; CK14, Thermo Fisher, Concentration (1:200), Cat# MA5-11599; RRID:AB_10982092; NCAM1, Abcam, Concentration (1:100), Cat# ab215981; ECAD, Abcam, Concentration (1:100), Cat# ab201499; ERG, Abcam, Concentration (1:50), Cat# ab214796; CD31, Abcam, Concentration (1:100), Cat# ab218582; RRID:AB_2857973; CD3, Concentration (1:100), Abcam, Cat# ab213608; Chromogranin A, Concentration (1:100), Abcam, Cat# ab215276; AR, Cell Signaling Technology, Concentration (1:100), Cat# 8956; RRID:AB_11129223; Alexa Fluor 647 AffiniPure Goat Anti-Rabbit IgG (H+L), Jackson ImmunoResearch, Concentration (1:500), 111-605-144; Alexa Fluor 488 AffiniPure Goat Anti-Mouse IgG (H+L), Jackson ImmunoResearch, Concentration (1:500), 115-545-003.
Validation	Please see the RRID IDs for each antibody used in the study. NRXN1 has been validated by Western Blotting in mouse brain tissue lysate and Immunohistochemistry (IHC) Analysis: A 1:1,000 dilution from a representative lot detected Neurexin-1 in mouse, rat, and human cerebral cortex tissue sections by EMD Millipore. NLGN1 antibody has been validated by Western Blotting in rat brain tissue lysate. IHC Analysis: A 1:50 dilution from a representative lot detected Neuroligin-1 in human cerebral cortex, rat cerebellum and rat cerebral cortex tissue by EMD Millipore. We validated both NRXN1 and NLGN1 antibodies using brain (positive control), colorectal (negative control) and prostate cancer tissue-sections in addition to PC3 and LNCaP cell lines (Supplementary Figure 11). Our results are shown in Figure 5 and Supplementary Figure 11. CK5 is validated using basal cells of normal human skin epidermis by BioLegend. CK8 is validated for IHC/IF using HeLa cells by Abcam. CK14 antibody has been validated using A-431 cells, squamous cell carcinoma of lung, mouse mammary glands and cultured P3 CD105+K14+ cells in various mediums in addition to advanced validation by cell treatment. NCAM1 is a recombinant antibody that has been tested for IHC/IF using U87MG cells by Abcam. ECAD antibody has been validated for IHC/IF uisng HT-29, MCF7 and BeWo cells, mesenchymal cancer cells, breast carcinoma, human colonic carcinoma, human papillary carcinoma, transitional kidney cell carcinoma of kidney tissue and human lung carcinoma by Abcam. ERG antibody has been validated for IHC/IF using human kidney, prostatic adenocarcinoma, GEDI-captured circulating tumor cells and THP-1 cell line by Abcam. CD31 antibody has been validated for IHC/IF using normal human kidney tissue sections by Abcam. CD3 antibody has been validated using human spleen, normal tonsil, normal spleen, normal brain, skeletal muscle and kidney (negative control) by Abcam. Chromogranin A has been validated for IHC/IF using human pancreas tissue and SH-SY5Y cells by Abcam. AR antibody has been validated using LNCaP cells by Cell Signaling Technologies.<br><br>In addition to the validation statements available through the manufacturer's website, we tested each antibody using fixed cell lines and FFPE tissue sections (Supplementary Figure 11, data available upon request). We also used Human Protein Atlas as a resource for antibody validation (https://www.proteinatlas.org/).

Eukaryotic cell lines

Policy information about cell lines

Cell line source(s)	PC3 (ATCC CRL-1435); LNCaP (ATCC FGC CRL-1740)
Authentication	Both PC3 (ATCC CRL-1435) and LNCaP (ATCC FGC CRL-1740) cell lines were authenticated using STR profiling.
Mycoplasma contamination	All cell lines tested were negative for mycoplasma contamination.
Commonly misidentified lines (See ICLAC register)	No commonly misidentified cell lines were used in the study.

Human research participants

Policy information about studies involving human research participants

Population characteristics	Samples were obtained from men with prostate cancer diagnosed in years 2004-2019 and who were treated with radical prostatectomy. Subjects age ranged from 46-81 years (median 64 years).
Recruitment	Samples were accrued with informed consent either through the (1) OHSU Knight Cancer Institute (KCI) BioLibrary; a tissue repository (OHSU IRB#4918) that provides deidentified human biospecimens and associated metadata, or (2) a separate study (OHSU IRB#18321) designed to investigate prostate cancer MRI, histology findings, and molecular characterization of tumors among men undergiong radical prostatectomy (PI Kopp). Patients were not compensated for participation in both studies. In both of these settings, prostate cancer samples were provided from men who underwent radical prostatectomy thus elected and/or were selected for surgery; thus there is potentially some bias towards younger, healthier men with prostate cancer compared to men who undergo radiotherapy. However, the oldest man represented was age 81. There may also be a bias towards larger, more clinically significant tumors among the lower risk group as these men were not selected for active surveillance.
Ethics oversight	Oversight is provided by the OHSU IRB 4918 and 18321.

Note that full information on the approval of the study protocol must also be provided in the manuscript.
